# Programs of Access to Pedagogy: Diagnostic of Its Design in Chilean Private Universities

**DOI:** 10.3390/ejihpe12040026

**Published:** 2022-03-25

**Authors:** Karen Núñez-Valdés, Neliot Villena Olivares, Cristian Villegas Dianta, Marisol López Núñez, Antonio Castillo-Paredes

**Affiliations:** 1 Escuela de Educación, Facultad de Educación, Universidad de Las Américas, Santiago 8320000, Chile; cvillegas@udla.cl (C.V.D.); mlopezn@udla.cl (M.L.N.); 2 Escuela de Pedagogía en Educación Física, Facultad de Educación, Universidad de Las Américas, Santiago 8320000, Chile; nvillena@udla.cl; 3 Grupo AFySE, Investigación en Actividad Física y Salud Escolar, Escuela de Pedagogía en Educación Física, Facultad de Educación, Universidad de Las Américas, Santiago 8320000, Chile; acastillop85@gmail.com

**Keywords:** Access to Pedagogy Programs, educational policy, initial teacher training

## Abstract

The objective of this study is to describe and analyze comparatively the Programs of Access to Pedagogies (PAP) of Chilean private universities, in order to know how this policy has been developed in this type of institution. After an exhaustive and meticulous treatment of the information contained in the 11 private university programs in Chile, categories were created for later analysis, based on the criteria contained in the programs. The results were analyzed based on pre-established analytical criteria, within which three to seven categories were found that allow the description and analysis of the PAPs. Among these, categories related to “Development of skills”, “Pedagogical vocation”, “Promotion of equity”, “Academic performance”, “Transversal skills” and “Academic support” stand out, being found in eight programs of the 11 analyzed. Although the Programs of Access to Pedagogy careers, established objectives, eligibility criteria, permanence criteria, camping, among others, follow the law, these programs have limitations, due to their heterogeneous construction, which translates into the interpretation of the laws, for which a series of challenges emerge, all of them associated with the search for strategies to strengthen the attraction and training of future good teachers.

## 1. Introduction

The quality of education and the different elements involved in it have been a constant concern of different governments in recent decades. The focuses of discussion have been varied and have referred to topics such as teaching and learning processes, teachers, teaching methodologies, evaluation, among others, contributing to the reflection on how to improve and strengthen education in the different levels. There is consensus that one of the elements that has a direct relationship with the quality of education is the teachers, since these are a relevant factor in the educational process, since their performance directly impacts the learning of their students, i.e., a good teacher achieves more and better learning [1,2,3]. Given this relevance, in Chile, a series of educational policies have been implemented whose objective has been to improve the quality of education from the teaching staff, considering their training and subsequent performance in the classroom, in order to overcome the gaps and inequalities that have historically persisted in the Chilean educational system [4]. Notable among these policies are Law No. 19,961 on Teacher Evaluation [5], Law No. 20129 that establishes a National System for Quality Assurance in Higher Education [6] and Law No. 20903, which creates the National System for Professional Teacher Development [7]. The latter, after 2016, focuses on improving the quality of initial teacher training (FID, by its acronym in Spanish) through different measures, such as the accreditation of pedagogy careers under certain standards and higher requirements for admission to these, in order to attract the best candidates for the teaching profession [8]. These mechanisms are aligned with the relevance attributed to teachers today. Similarly, this law establishes mechanisms to attract outstanding high school students who show interest and vocation towards a teaching career, so that universities can design and implement devices that promote access to pedagogies in an equitable and inclusive manner [9]. Among these devices are the Pedagogy Preparation and Access Programs, whose main objectives are to “Increase equity, diversity and quality in higher education, specifically, in pedagogy careers and programs, as well as generating new perspectives in secondary education” and “early capture students with interest and conditions to exercise the functions of education professionals in educational institutions” [10]. These programs must be elaborated and presented by each university for the recognition of the Ministry of Education, being relevant for its approval the description of the strategies that are contemplated to prepare the students for their entrance to the pedagogy careers. Consequently, there are a variety of offers of this type of program since each institution designs these according to its institutional purposes and educational model. There is a history of the characteristics of the Access Programs in the universities depending on the State of Chile, given in the article by [9], but there is no information regarding how these programs have been designed in private institutions in the country; a series of questions arise regarding these articles: How are the programs taught in Chilean private universities configured? What are the emphases that these universities give to their programs? Are there common characteristics between the programs of these universities? Given these questions, this study has been proposed to describe and analyze in a comparative way the Programs of Access to Pedagogies (PAP) of the Chilean private universities, to know how this policy has been developed in this type of institution.

With this research, it is expected to contribute to the knowledge about the Pedagogy Access Programs (PAP) and how this Chilean educational policy is configured in higher education institutions, specifically in private universities. This is considering that there are no studies associated with the subject in this type of university. With the results presented, it is hoped that readers will have precise and detailed information on how PAP programs are articulated in Chilean private universities, so that, with it, they can make decisions that allow improvements in the implementation of this educational policy.

### Theoretical Framework

In the last fifteen years, and after the accreditation processes of pedagogy careers, the initial teacher training models have been questioned due to advances in the field of knowledge construction and interpretation. Similarly, teacher training has been raised as a key factor in improving the educational system to narrow the persistent gaps in it [11], being relevant its analysis to improve these, because a good teacher is the most important predictor of student performance within the school system [12].

The results obtained in the first years of the initial test showed deficiencies in training, where the teachers in training were not better than those in the educational system [13], thus revealing that the State did not have the instruments and policies that would ensure the quality of initial training in Chile. These data initiated a series of transformations that have tended to strengthen public policy on initial teacher training (FID), especially with regard to a quality teaching profession. Within the framework of these transformations, the attraction and selection of students interested in studying a pedagogy career has been enhanced, as well as an increase in the requirements associated with the ranking of high school grades or the score in the university selection tests [7]. Along with this, Law No. 20,529 of 2011 on the Quality Assurance System [14], the development of the Framework for Good Teaching, the appearance of the Guiding Standards for Initial Teacher Training, the Curricular Bases, the accreditation criteria of pedagogy careers and finally the creation of the Teacher Professional Development System, supported by a series of laws associated with the New Public Education and School Inclusion, have formed a system that demands a solid initial teacher training program for the country, thus leading to a series of challenges to achieve the required quality.

Currently, there are some critical issues such as the deficient level of skills for entering pedagogies [15], where the application of diagnoses and their corresponding remedial actions have not been able to fill the gaps that the educational system shows. The lack of sufficiency in the quality of training programs, the deregulation of programs, non-specialized training for the teaching of disadvantaged social groups and the academicism of the disciplines [11] are some of the issues that initial teacher training must address with urgency. Another critical issue is the lack of a national reference framework that fosters coherence between the different pedagogy programs existing in the country and the professional performance that is requested, in addition to the projection of a deficit of educators due to the increase in selectivity in the admission to pedagogy careers [16].

In this way, the legislation in Chile creates a mechanism for the assurance of quality in education. The Real Academia Española (RAE) defines quality as “property or set of properties inherent to something, which allow judging its value” [17]. From a philosophical point of view, quality has determinations of compromising its ontological environment and, in the transition to education, is based on rules that intervene in the training process at an individual and social level, deepening dimensions and indicators for the development of conditioning cognitive processes for professional performance in particular sociocultural, ideological and economic contexts of a population in general in a given context [18].

A determining element in the quality of the educational system is the coherence between the design of public policy as well as its implementation and the results obtained [19], which has become evident in recent decades in initial teacher training, which has been characterized by: (a) a bureaucratized training, (b) the separation between the theory and practice, (c) the fragmentation of knowledge and (d) the lack of a link with the school [20], which make current training insufficient and outside the criteria of the National Education Commission. In the same way, it is necessary to address the new challenges that emerge, such as those set forth in the new initial training standards for the year 2021, since, in these, it is evident that, beyond specific knowledge, it is necessary to advance in the attention to the development of socio-emotional, affective and in the citizenship education of the students [21], thus aiming at a comprehensive education of the future teacher. In this context, early and progressive practices that allow students to build processes of identification with the profession have been strengthened [22], but it is still necessary to deepen the construction of professional identity and direct work with the classroom and the system. Another of the challenges of initial teacher training is the need to attract students who have better academic and vocational conditions that can be projected in a future professional performance in order to raise the quality of training and thus have evaluative standards for training programs [23]. These elements that seek to enhance initial training must dialogue with the educational gap of the school system, such that, in higher education, programs of access to pedagogies have appeared as an alternative of special income independent of the University Selection Test (PSU, by its acronym in Spanish) score and the grade point average required by current legislation to access pedagogy careers [24].

In this context, in 2016, Law No. 20,903 [7] was enacted, which creates the National System of for Teacher Professional Development, which incorporates within its objectives the quality assurance of initial teacher training, support for the job placement of education professionals and the permanent development of training policies for professional development. Among the specific aspects of this law, it is established that universities may design and implement special access programs to pedagogy, where the main objective is to allow students with an interest in the teaching profession and with glimpses of pedagogical vocation to join said programs to strengthen their skills and enter to study pedagogy through this entry route [7]. This path is based on the idea that there are many students with talent and concern or interest in pedagogy, who often do not meet the entry requirements for this type of career but do show a predisposition to teaching and a good school academic career, so these programs will allow the inclusion of students from different contexts and of educational establishments of different dependencies. In this way, the preparation and access programs for pedagogy careers are aimed at high school students who would like to study pedagogy and who meet a series of requirements according to the university that dictates the program [9]. Students who pass this type of program can access the pedagogy careers that are included in each program, after having taken the Transition Test (PDT, by its acronym in Spanish), regardless of the score they obtain in the selection test or the ranking of grades they obtained by the end of high school. The programs include pedagogical training modules, where the vocation of the students is developed. In addition, activities of a cultural nature and academic levelling are carried out, which aim to ensure that students are successful in entering higher education [25]. For the aforementioned reasons, it is that universities can apply for this type of program before MINEDUC, which is in charge of endorsing them for their operation. The Department of Institutional Strengthening of the Ministry of Education (FID) is in charge of approving, guiding and accompanying universities in the implementation of this type of program. The FID provides the general guidelines for the development of the programs, but there is no single type of these, since each university defines the profile of students, the academic modules to be carried out and the management, among other aspects, so it is necessary to make a review and comparison of the currently available programs, to identify and analyze the differences between them.

In this way, there could be the possibility that these access programs integrate the requirements of the legislation in Chile [5,6,7,8]; however, is it possible that they fully integrate the social, political and theoretical requirements for the training of a quality teacher? The literature indicates that a good teacher must possess competencies inherent in teacher training, such as research, management, participation in accreditation processes, pedagogical competencies and reflection on their educational process in teacher training [26]. In addition, it must have characteristics of human nature and some characteristics that are consolidated through the exercise of the teaching profession [27], since education must contribute to happiness, well-being and social resilience [28], to favor the construction of a better society and equal rights and opportunities [29].

## 2. Materials and Methods

In this section, the procedures and the method chosen for the development of this study will be detailed.

In this research, a qualitative methodology was used. The choice of this methodology is due to the proposed research objectives, opting for documentary analysis, which has the purpose of representing the information of a certain document in a synthetic, structured and analytical way [30]. This type of analysis makes it possible to describe and represent the content of the documents in a schematic and unified way, in order to evaluate their underlying information [31].

In this study, through an analytical–comparative process, the presentation forms to the Ministry of Education of the Programs of preparation and access of secondary students to continue studies of pedagogy in higher education of 11 private universities in Chile were analyzed. An analysis of the common criteria was carried out in the dimensions of design, implementation and global evaluation of the programs, which are enshrined in the presentation form, namely objectives, admission requirements, diagnostic mechanisms, preparation modules, management model, program evaluation, student enrolment at the university, support systems, links with other programs and support of students at FID. The analysis developed was based on the comparative systematization of the forms, where both common patterns and differentiating patterns were identified between programs.

The information was processed in the Atlas Ti 9 program, where emerging codes/categories were raised in each of the predefined criteria, which have been reported in terms of frequencies or their content. The presentation of the results is structured based on the elements that make up the Access to Pedagogy Programs.

The limitations of this study and the methodology used are linked to the fact that only the presentation forms to the Ministry of Education were analyzed and not other elements, such as the results of the participating students, the opinions of their managers, among others.

## 3. Results and Discussion

In this section, the results obtained after the development of the analytical process will be presented, which will be accompanied by a discussion around them.

The presentation of the results and the discussion of these have been carried out based on the previously established criteria structure of the Access to Pedagogy Programs and the categories found in the analytical process; therefore, the names of the programs or the universities analyzed are not identified. Below is a summary table with this information and then each category with its respective analysis (Table 1).

### 3.1. Objectives of the Program

In the training programs, various types of objectives are noted, which have been grouped into categories. Six categories have been raised, which account for the emphasis that each program has. The most recurrent categories are those associated with the pedagogical vocation of students and the promotion of equity. Those programs in whose objectives the pedagogical vocation is relieved agree that among their purposes are promoting the access of those students who express interest in starting a pedagogical career; as an example, one of the stated objectives is “To promote the attraction, access and permanence of young people with vocation and pedagogical talent”. The need to have students with a vocation lies in the fact that this is a characteristic that stands out in professional practice, since teachers with a vocation can be classified as people with suitable characteristics to develop and practice teaching [32]. Equally important is the promotion of equity through access to these programs, since it is expected that students who enter them will have the possibility of studying a pedagogical career despite their social context and the gaps that this could generate. One of the programs has as its objective “Promote equity in access by shortening gaps typical of socioeconomic and cultural differences in the results generated by PSU and Grades Ranking”. The programs also hope to develop skills in areas considered a priority for university access, such as logical mathematical thinking and the production of oral and written texts, ultimately betting on the levelling of the skills associated with these. Likewise, the objectives refer to the importance of developing personal skills necessary for professional training, namely self-regulation and autonomy. To a lesser extent, the programs mention in their objectives the improvement of the quality of education, this being a minor but no less important reference to point out. One of the programs that alludes to this category states in its objective “In addition, it aims to promote equity in university access, and favor their permanence and graduation through the comprehensive training of the student in the areas of knowledge, procedural and value, to enter the world of work as quality teachers, with special emphasis on the teaching vocation”.

Table 2 shows the frequencies of the categories that emerged from the objectives presented by each of the programs. This shows the relevance of the pedagogical vocation of the students and the promotion of equity.

### 3.2. Admission

The admission processes of the analyzed programs refer as entry requirements to academic performance, the interest in pedagogy, the development of an interview, the students’ belonging to institutions with an agreement with the university, participation in activities defined by the program, the recommendations of the institution of origin and the commitment expressed by the students. Below are the frequencies of the categories of this analytical criterion (Table 3).

In these criteria, the programs focus on “academic performance”, i.e., they give greater importance for admission to the performance that applicants have had in their respective educational establishments, which is aligned with the requirements for admission to pedagogy careers, since Law 20903 gives relevance to the average of high school grades. The study [25] indicates that there is an important relationship between the previous academic performance of students entering higher education and their academic performance at the university since those who perform well in school are more likely to achieve good performance in higher education. In the work of [33], it is highlighted that in-service teachers survey the academic performance of their students along with social participation and leadership, as aspects to consider when selecting the best candidates for a teaching career. The criterion “interest in pedagogy” has a close relationship with a philosophical view regarding “vocation”, because it allows reflection on the production of a change in social, educational and cultural communities. In turn, the criteria “interviews” and “student commitment” allow the programs to carry out a qualitative survey on the students, since relevant evidence is collected on how the students see their entrance to the university and in particular to a career of pedagogy. The inclusion of the commitment is an input for institutions on how the student perceives his entry into this type of program and how they eventually manifest their interest in pedagogy. One of the institutions refers to this point: “Student Commitment Letter: this letter declares student’s intentions to participate in the Propaedeutic program and the vocational interest in studying a pedagogical career”.

### 3.3. Diagnostic Mechanisms

**Figure 1:**
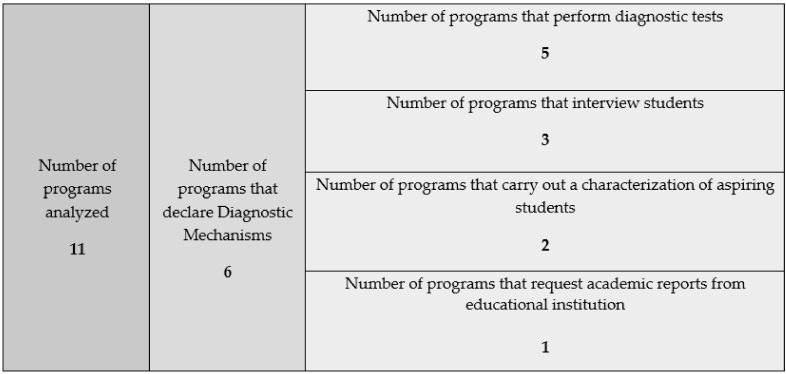
Diagnostic mechanisms. Source: Authors’ own creation elaboration.

Concerning the diagnostic mechanisms of the access programs carried out on their students, six institutions that declare them were detected. Of the six institutions, five of these base their diagnoses on tests, three indicate that they conduct interviews with students, and one requests academic reports from educational establishments. It is relevant to mention that two programs perform a characterization of the students who participate in the program, as they refer to a desire to “know the admission characteristics” (Figure 1).

Some institutions encourage direct entry to the program after selection, so the student takes an assessment after being admitted (diagnostic test), while, in other cases, the program has a propaedeutic stage, which, once approved, allows students to take the assessment. From the analyzed institutions, some of these conduct individual and group interviews as diagnostic mechanisms, whose results allow them to identify the gaps and challenges presented by each cohort of students, which for [34] is fundamental to attract good students to the teaching profession, train them properly and retain effective teachers. Regarding diagnosis themes, those associated with the pedagogical skills and potentialities of the students predominate, addressing topics such as professional vocation, competencies for university life, reasoning and learning styles, while other institutions integrate the evaluation of communicative skills, general skills, interpersonal relationships and mathematical reasoning. The latter is included mainly because it leads to formal reasoning, thus allowing problem-solving, decision-making, analysis, synthesis, the elaboration of conclusions, among others [35].

### 3.4. Preparation Activities and Modules

Regarding the curricular structure of the programs, eight of the eleven institutions analyzed indicate that they work on the development of transversal skills, ten programs carry out courses or workshops associated with the development of the pedagogical vocation and seven programs implement modules related to personal management. It is important to note that the inclusion of transversal skills in a large number of programs is justified by current trends, since, according to the World Economic Forum, a third of all skills for future job performance will be associated with emotional intelligence, social relationships and teamwork [36].

One of the programs gives great importance to music education, as well as others to the development of linkage activities and extension activities. An example of the activities carried out by the programs is stated by one of these: “Conversations are held with academics of the career of their interest, as a way to get to know their future teachers and the topics addressed by each discipline. Participation in activities of the careers such as attendance at some of the classes, academic presentations, inclusion fairs and all those that are part of the curriculum. In that instance, they will be able to share the development of university teaching-learning activities with students from various careers.” This type of activity allows students to develop connections with the university community, since it is from these that experiences and opinions can be acquired [37].

Other institutions, specifically two, declare the existence of workshops but do not specify the theme of these and their relationship with the objectives of the program.

Within the curricular activities that are developed, some programs aim to enhance transversal skills such as mathematical thinking, argumentation, emotional development and autonomy, while other programs are aimed at the development of modules based on the school curriculum, such as communication and math.

The coincidence between the programs on the format of the preparation activities/modules is relevant. Some are courses in a workshop mode, where constant dialogue and attention to the training needs of students is declared. This shows that the programs try to get their students to use dialogue as a learning tool, which is very typical of educational practice, since it is from this that students can be induced to think and reason, express ideas and question different propositions [38].

### 3.5. Management Models

In relation to the management model, most of the access programs declare the articulation with different units belonging to the Academic Vice-Rectory of the universities that teach them. On the other hand, it is declared that the programs are dependent on the Faculties of Education and/or Humanities, represented by the figure of the Dean. One of the programs states about its management model: “The Access to Pedagogy Program is based in the Department of Pedagogy of the Faculty of Education Sciences. The department has a presence in all the headquarters and a structure that supports the different careers. The department liaises with the teams at each venue responsible for the implementation of the program, which includes the outreach, venue, and careers teams. In each campus, there is an Academic Director who depends on the Academic Vice-Rectory and the Vice-Rectory of Headquarters in an organic matrix structure. At the national level, the National Director of the Department relates to both, the Academic Vice-Rectory, the Vice rectory for Student Affairs and the Institute of Student Performance and Support”. A smaller number of universities declare that there is an executive team for the program, represented mainly by the figure of a program coordinator. Among the functions of this position are “to organize the academic team of the program, coordinate the academic lines of language, mathematics, social sciences and technology, and the training and recreational lines of the program. The coordinator has the responsibility of participating in the processes of creation and evaluation of subjects programs, graduation profile and curriculum. In addition, it must carry out a process of monitoring/accompaniment of the progress of the students once enrolled, during the first two years of training”. Some universities have other types of propaedeutic programs, which are articulated with the programs of access to pedagogy and share the management model for both instances. Finally, according to what has been observed, it is possible to point out that the programs articulate with different units according to the internal organization of each university, which is responsible for managing the different aspects of the implementation of this type of program.

### 3.6. Graduation and Enrolment of Students at the University

**Figure 2:**
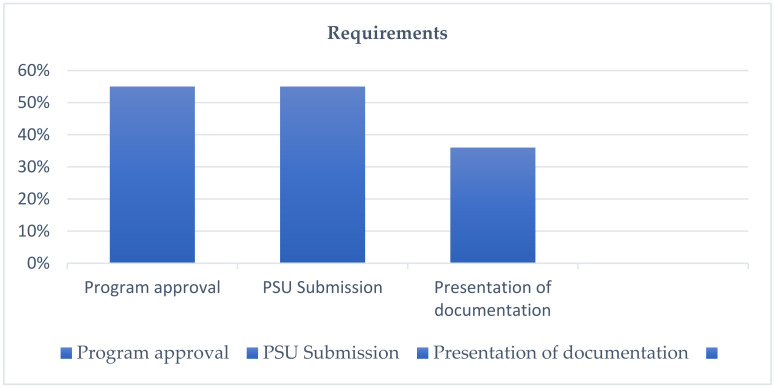
Requirements for graduation and enrolment of students in the university. Source: Author’s Own creation elaboration.

Regarding graduation and student enrolment at the university, six programs declare components related to program approval and the submission of the PSU (Figure 2). Both criteria are relevant in higher education, since the approval of the program will allow the levelling of theoretical, practical knowledge and socio-emotional skills, taking into account that the law establishes admission to higher education careers through a standardized test. In this regard, one of the programs states: “Enrolment requirements and conditions enabled: the requirements requested to the student to access a vacancy in any pedagogy career are: either having taken the PSU or having 100% attendance at the program (with exceptions duly justified such as medical causes or other emergencies)”. In addition, four programs declare components related to documentation, which are relevant to both admission to the program and the university. The student must comply with the documentation that proves the completion of their high school training process, as well as the presentation of the documentation of approval of the program, identity documents, medical documents, PSU submission and Unique Socioeconomic Accreditation Form (FUAS, by its acronym in Spanish) registration.

### 3.7. Accompaniment Systems

The programs must declare accompaniment and levelling mechanisms that the institution will provide to the students who will enter higher education and who have been part of them. Of the programs analyzed, there are coincidences in the accompaniment strategies that will be deployed, since the universities refer both an academic and a personal accompaniment to students entering the first year. Regarding the first type of accompaniment, one of the universities indicates that “it will provide academic support to the student, through the Learning Centres tutoring system, academic assistantships, and workshops on learning and basic science, and performance of critical subjects”. Universities agree to provide students with tutorials, assistantships and/or workshops as a form of support in the first year of university. These strategies contribute to the retention and permanence of students in higher education since the support of the institution will allow them to develop and enhance their abilities to function in the different scenarios in which they have to interact [39,40,41].

Regarding the personal accompaniment, each university grants its own distinctive mark; for example, some institutions refer to a personalized follow-up: “monitoring will be carried out through an individual follow-up form, instrument that will be monitored by the programs coordinator, and that will integrate academic and psychosocial information from the students”. Meanwhile, other institutions give this accompaniment a socioeconomic approach, referring to it as “personal and socioeconomic accompaniment: social worker makes home visits, to know the context that the students live and their socioeconomic condition”.

The declared accompaniment strategies aim to provide the conditions and services necessary for the success of students in their university life, which is part of the important efforts that higher education institutions around the world make to ensure the permanence and academic success of their students [42].

### 3.8. Program Dissemination Mechanism

Among the diffusion mechanisms for these programs, the agreements with educational establishments stand out, which are declared by seven out of the eleven analyzed institutions. Another of the diffusion strategies adopted by the universities is the relationship with different municipalities, which, through their education departments, centralize the invitations to participate in these programs. An example of this strategy is: “Agreement between the municipality of Peñaflor and Lampa, in addition to other educational establishments beyond these municipalities”. In this way, universities are linked to their environment through educational institutions, both municipal and subsidized–private and the different municipalities that open their doors to the development of diffusion activities. This broadcasting strategy is aligned with one of the missions of the university, which is the link with the environment, understood as the set of links between higher education institutions and the social environment in which they are inserted to respond to the demands that society has [43].

### 3.9. Accompaniment of Students at FID

As for the criterion accompaniment of students in the FID, all universities declare instances of academic support for students who enter to study pedagogy; as an example, a university indicates the realization of “levelling of contents prior and during classes, workshops, academic mentoring, accompaniment of facilitator par and academic follow-up”. On the other hand, support appears at the psychological and psycho-pedagogical level, through work teams or psychologists who support students in the course of their careers. Another aspect mentioned in the programs is the support in the integration to university life, an aspect that is not detailed in the documents, but it is presumed to have a comprehensive nature and not just an academic focus. Finally, one aspect mentioned is the application of diagnoses that would allow universities to see the progression of students during the development of their university career (Table 4).

Due to the amount of information, support instances and the level of actions indicated by the different programs, it is possible to infer that this aspect is essential for universities, and there is a commitment to initial teacher training. The support strategies referred to by the institutions analyzed are recognized by the literature for their positive impact on students, since they contribute to an increase in academic performance and therefore to student retention, permanence and timely graduation. In the same way, they have been promoted by the Ministry of Education, which has allocated resources to finance some of these strategies, such as the Academic Levelling Scholarship [39].

### 3.10. Practical Implications

Initial teacher training in Chile is based on politics, legislation and the actors that make possible the process of professional improvement for an improvement in educational quality [15]. From a legislative and professional perspective, the results of this research show an interpretation of the legislation, politics and sociodemographic reality of Chilean private higher education institutions. There are nine major analytical criteria related to “program objectives”, “admission”, “diagnostic mechanisms”, “preparation activities and modules”, “management model”, “graduation and enrollment of students in the university”, “accompaniment systems, “program dissemination mechanisms” and “accompaniment in the FID”, based on their respective categories.

Although the accompaniment programs have analytical criteria and common categories, it is expected that in their construction, there will be their own categories associated with the different pedagogy careers and the interests of the future professional, based on historical factors, theoretical aspects of knowledge, knowing how to be and knowing how to do.

### 3.11. Future Directions

Based on the findings of this research, it would be relevant for the university institutions to consider that, within the content, activities and internal mechanisms of the programs, there are specific categories of each pedagogy career [41], such as Pedagogy in Basic Education, Pedagogy in Differential Education or Pedagogy in Physical Education, since, although these are pedagogy careers, each of them has particular characteristics in the exercise of the teaching profession, which could serve to strengthen the teaching vocation and contribute to emotional education [44].

In addition, these access programs accompany the future pedagogy student from the penultimate or last year of secondary education; however, it would be interesting for the institutions to consider carrying out the accompaniment from the beginning of secondary education, progressively allowing the development of a pedagogical vocation and the accompaniment of higher education institutions in school training processes.

Finally, it is suggested as future lines of work to investigate the success of this type of program and how they contribute to the development of a pedagogical vocation in students prior to entering higher education. In the same way, it is suggested to investigate how the characteristics of a certain institution influence the development of this type of program and the configuration of the pedagogical vocation.

### 3.12. Limitations

One of the main limitations of this study is the impossibility of determining the percentage of success of these programs, taking into account the entry, permanence and completion of high school students in the chosen pedagogy career. On the other hand, these programs have been designed based on what was requested by the Ministry of Education of Chile; however, when analyzing the characteristics of each one of these, it is evident that some highlight the characteristics of the institution that presents it and of the type of pedagogy they teach, as is the case of a program where Music Education has great importance for its development, which has not been analyzed in this study and could be an important element to consider in future studies.

## 4. Conclusions

The admission of students to pedagogy careers must be carried out beyond the results obtained in the University Selection Test (PSU, standardized test of access to Chilean universities) and the Ranking of High School Grades since both mechanisms have limitations; that is, they do not allow the detection of skills, talents, qualities, vocation and interests in university applicants. Detecting these skills acquires great relevance since they influence the training of good teachers, recognizing their impact on the learning of students and therefore on the quality of education [1,2,3]. In this context, the Programs of Access to Pedagogies (PAP) are an alternative way of entering higher education and are constituted as a strategy where the interest of students in pedagogy is valued [45], so knowing the characteristics of these in a heterogeneous educational system such as the Chilean one is an input to outline paths of improvement and glimpse the challenges for access to pedagogy careers.

From the programs analyzed in this study, it can be deduced that their objectives are mainly based on seeking pedagogical talents who have a vocation for careers in this area, with the hope that the students will be able to start a pedagogical career. Pedagogical talent and vocation are considered as elements that influence the training of good teachers and their subsequent professional practice [32], so these programs are expected to strengthen the attraction, access and permanence of the students with a profile that approximates the basic characteristics of a good teacher; it is expected to reduce the access gaps due to the socioeconomic and cultural conditions of the applicants.

Concerning admission mechanisms, great importance is given to the academic performance of the applicants, a situation that aligns with the predictability of this variable in the performance and permanence of a student in higher education, which, according to various studies, is related to the successful performance of these in their university education [23,33]. It can be concluded that the purpose of the admission mechanisms is to attract students with high academic performance in their respective educational institutions and to express the interest in studying a career in pedagogy.

The diagnostic mechanisms defined by the different programs are concentrated in two areas: on one hand, there are those mechanisms of a qualitative nature, such as interviews; on the other hand, there are those of a quantitative nature, associated with the application of tests. The execution of these depends on how this process has been defined within the program since some prefer one mechanism over the other, while there are programs that apply both.

The programs define preparation modules for accepted students; these are preferably carried out in workshop format and use a dialogic methodology—that is, it is through dialogue that the teaching and learning process is developed. This methodology favors the development of communication skills, critical reflection, participation, empathy, among others, thereby increasing student learning [45]. In addition, the programs incorporate the development of courses/modules of transversal skills, such as the development of mathematical logical thinking, which allows the acquisition of abstract knowledge, reasoning and judgment of relationships between different concepts.

The management models of the programs are based on the articulation of these with different organizational instances of the university, all linked to the Academic Vice-Rectory of each institution. With this, the development of collaborative work between the different units involved is expected in order to take advantage of institutional resources in pursuit of the achievement of the objectives of the program and the institution. Regarding the admission and enrolment in the university of students who belonged to an Access Program, this is mainly conditioned on the approval of the program and the submission of the PSU, being the requirements that all programs declare.

The accompaniment strategies that the universities carry out for the students who were part of a program and later enrolled in a pedagogy career are both academic and personal, which is due to the monitoring mechanisms that the universities use to ensure the retention and permanence of students in the institution. This has become especially relevant in recent years since both retention and timely graduation are indicators of academic management in the accreditation processes [39].

As for the diffusion mechanisms used by the universities to publicize their programs to the community, the most used are the agreements with educational establishments, since the institutions address them directly to summon the possible students interested in participating in the programs. Another mechanism is the link with municipalities, where the callings to participate in the programs are displayed. Both mechanisms obey a form of connection with the environment, this being essential for universities to meet the needs of society [43].

Finally, the accompaniment of students in their FID is part of the interest of keeping them in the university, and in their retention, permanence and timely graduation, developing different strategies for it, such as sports and recreation workshops, psychological support, scholarships and financial aid, academic levelling, tutorials, assistantships, among others. All these strategies are intended to contribute to the follow-up of students to ensure the realization of their university education [36].

In short, the analysis of these categories provides us with accurate and detailed information on how Chilean private universities have configured their PAP offer. This document has considered the objectives, admission, diagnostic mechanisms, activities and preparation modules, management models, graduation and enrolment of students to universities and the system of accompaniment in the FID. The convergences and divergences of the programs of private universities that are currently active have emerged and present a challenge as we seek strategies to strengthen the training of future pedagogues.

This article has been limited to the theoretical description of the PAP in force in Chilean private universities, so investigating their development and effects is a task for future research. However, it is expected to contribute to the knowledge of these to promote their continuous improvement and thus provide evidence of the implementation of educational policy.

## Figures and Tables

**Table 1:** Summary of analytical criteria and emerging categories.

Analytical Criteria	Categories
Objectives of the Program	Skills development Personal development Pedagogical vocation Equity promotion Accompaniment Improving the quality of education
Admission	Academic performance Interest in pedagogy Interviews Agreements with the institution Participation in artistic activities Recommendation of the establishment Commitment to the program
Diagnostic Mechanisms	Tests Academic reports Interviews Diagnostic evaluation Student characterization
Activities and Preparation Modules	Transversal skills Pedagogical vocation Personal management Music education Linking activities with the environment Outreach activities Workshops
Management Model	Institutional articulation Advisory board Executive team
Graduation and Enrollment of Students in the University	Adoption of the agenda Registration in FUAS PSU Documents
Accompaniment Systems	Counseling Leveling programs Academic accompaniment Personal and socio-economic accompaniment Follow up
Program Dissemination Mechanisms	Town halls Agreements with educational establishments Collaborating educational establishments PACE schools Broadcast
Accompaniment of Students at FID	Academic support Support for integration into university life Diagnostic evaluation Psychological and/or psycho-pedagogical support Satisfaction surveys

Source: Authors’ own creation elaboration.

**Table 2:** Frequencies of the categories of the criterion objectives of the program.

Categories	Frequencies
Skills development	8
Personal development	3
Pedagogical vocation	10
Equity promotion	10
Accompaniment	5
Improving the quality of education	3

Source: Authors’ own creation elaboration.

**Table 3:** Frequencies of the categories of the criterion Admission.

Categories	Frequencies
Recommendation of the establishment	2
Interest in pedagogy	5
Interviews	4
Academic performance	8
Agreements with the institution	1
Participation in artistic activities	1
Commitment to the program	4

Source: Authors’ own creation elaboration.

**Table 4:** Frequencies of the categories of the criterion accompaniment of students at FID.

Categories	Frequencies
Academic support	11
Psychological support	6
Support in the integration to university life	4
Application of diagnoses	4

Source: Authors’ own creation elaboration.

## Data Availability

The documents analyzed were downloaded from the official page of the chilean Ministry of Education http://dfi.mineduc.cl/index2.php?id_seccion=5472&id_portal=59&id_contenido=34645, accessed on 15 October 2021.
